# Assessment of transparency and selective reporting of interventional trials studying colorectal cancer

**DOI:** 10.1186/s12885-022-09334-5

**Published:** 2022-03-15

**Authors:** Anna Pellat, Isabelle Boutron, Philippe Ravaud

**Affiliations:** 1 Gastroenterology and Digestive Oncology Unit, Assistance Publique Des Hôpitaux de Paris, Hôpital Cochin, 27 rue du Faubourg Saint Jacques, 75014 Paris, France; 2 Université de Paris, Centre of Research in Epidemiology and Statistics (CRESS), Inserm U1153, 1 Paris Notre Dame, 75004 Paris, France; 3 Centre d’Épidémiologie Clinique, Assistance Publique des Hôpitaux de Paris, Hôpital Hôtel Dieu, 1 Parvis Notre Dame, 75004 Paris, France

**Keywords:** Colorectal cancer, Interventional trials, CONSORT statement, Completeness of reporting, Data sharing

## Abstract

**Background:**

Colorectal cancer (CRC) is currently one of the most frequently diagnosed cancers. Our aim was to evaluate transparency and selective reporting in interventional trials studying CRC.

**Methods:**

First, we assessed indicators of transparency with completeness of reporting, according to the CONSORT statement, and data sharing. We evaluated a selection of reporting items for a sample of randomized controlled trials (RCTs) studying CRC with published full-text articles between 2021–03-22 and 2018–03-22. Selected items were issued from the previously published CONSORT based peer-review tool (COBPeer tool). Then, we evaluated selective reporting through retrospective registration and primary outcome(s) switching between registration and publication. Finally, we determined if primary outcome(s) switching favored significant outcomes.

**Results:**

We evaluated 101 RCTs with published full-text articles between 2021–03-22 and 2018–03-22. Five trials (5%) reported all selected CONSORT items completely. Seventy-four (73%), 53 (52%) and 13 (13%) trials reported the primary outcome(s), the allocation concealment process and harms completely. Twenty-five (25%) trials were willing to share data. In our sample, 49 (49%) trials were retrospectively registered and 23 (23%) trials had primary outcome(s) switching. The influence of primary outcome(s) switching could be evaluated in 16 (16/23 = 70%) trials, with 6 (6/16 = 38%) trials showing a discrepancy that favored statistically significant results.

**Conclusions:**

Our results highlight a lack of transparency as well as frequent selective reporting in interventional trials studying CRC.

**Supplementary Information:**

The online version contains supplementary material available at 10.1186/s12885-022-09334-5.

## Background

Cancer is currently an important public health issue worldwide. Colorectal cancer (CRC) is the third most commonly diagnosed cancer in males and the second in females. In 2020, more than 1.9 million new cases were diagnosed according to the World Health Organization Global Cancer Observatory (GCO) database (https://gco.iarc.fr/*).* In the past years, an increasing rate of interventional trials have been conducted in oncology [1, 2] in order to improve screening, find new treatments and overall improve prognosis and quality of life of patients with cancer.

Previous studies highlighted an important waste in the production and reporting of research in various fields [3, 4]. This waste could happen in the different research steps: inadequate research question, inappropriate study design, conduct and analysis, inaccessible research results and incomplete or unusable reports of study documentations and results [4, 5].

Lack of transparency and selective reporting of trials are common and main issues when it comes to interpretation and reproducibility of results [6–8]. In order to help with trial reporting, various guidelines have been developed for each type of research. For instance, the Consolidated Standards of Reporting Trials (CONSORT) statement issued reporting guidelines for randomized controlled trials (RCTs) in 2010. Furthermore, access to study protocols and documentations can help detect selective reporting such as primary outcome(s) switching [5].

## Methods

The aim of our work was to assess transparency through completeness of reporting and data sharing intention, as well as selective reporting, in RCTs studying CRC management.

### Search strategy and eligibility criteria

This work is a follow up study of a previous work aiming to assess availability of results in CRC trials. Our search strategy on the ClinicalTrials.gov registry has been previously described (*submitted article*). Details for our search strategy and eligibility criteria are available in Additional file 1.

We evaluated a sample of completed RCTs studying CRC management in adults, registered on ClinicalTrials.Gov, and with results published in a full-text article in English between 2021–03-22 and 2018–03-22.

### Data extraction

Data extraction was done by one independent reviewer. In case of difficulties, it was discussed with a senior reviewer. We developed a standardized data extraction form (Additional files 2 and 3). The extraction was done with all trial documentations available (full-text articles, protocols, statistical analysis plans and the ClinicalTrials.gov registry when appropriate).

### Outcome measures

#### Transparency indicators

##### Access to the trial documentation

We systematically checked whether we had access to the protocol and statistical analysis plan, and where (registry/article). We also looked at the content and determined whether we had access to the full protocol or to an abbreviated or redacted protocol and whether the protocol was available in English.

##### Data sharing

We recorded whether investigators had made a data sharing statement (availability statement), and if/where/how investigators planned to share data (retrieval methods, accessibility, content, date). We also assessed if the statement was done through the registry and/or through the published article (Additional files 2 and 3).

##### Completeness of reporting

Completeness of reporting of articles was assessed using a modified version of the CONSORT-based peer-review tool COBPeer tool checklist (Table 1) consisting of the 11 most important and frequently incompletely reported CONSORT items [9, 10]. Each item is elicited with sub-items explicating what should be reported. We evaluated for each sub-item if the information was reported: Yes/No/Not-assessable (NA). Finally, each item was rated as “completely reported” (i.e. if all sub-items were adequately reported), “partially reported” (i.e. if at least one sub-item was missing) and “not reported” (i.e. if all sub-items were missing). The overall trial reporting rate followed the same rule using each items’ final reporting result. If the primary outcome was not clearly defined in the full-text article, and in order to evaluate the CONSORT subitem 6a “reporting of primary outcome” (Table 1), we chose the outcome used for the primary objective, or for the calculation of the sample size, or the first primary outcome listed in the registry. If trials had several primary outcomes, we applied the same strategy for each primary outcome and rated the overall reporting.

**Table 1:** Modified version of the COBPeer tool [9]

CONSORT items and subitems					
**METHODS**					
**Outcomes** *Comments:* *3: The outcome assessment should be described using an existing scale or individually defined parameters* *5: Summary of measure needs to be given and specified for each primary outcome. General measurements for all outcomes are not valid* *7: Person who analyzed the outcome should be clearly identified*	**Item 6a. Completely defined pre-specified primary outcome measures, including how and when they were assessed** 1. Was(were) the primary outcome(s) clearly identified (e.g., the primary/main outcome was pain)? * **For each primary outcome evaluated, were the following elements reported:** 2. The variable of interest (e.g., pain, all-cause mortality) 3. How the outcome was assessed (e.g., VAS, Beck Depression Inventory score, pain scale) 4. The analysis metric (e.g., change from baseline, final value, time to event) 5. The summary measure for each study group (e.g., mean, proportion with score > 2) 6. Time point of interest for analysis (e.g., 3 months) *NA*^*a*^* if survival analysis* 7. Who assessed the outcome (e.g., the patient, doctor, nurse, caregiver, other)				
**Randomization/sequence generation**	**Item 8a. Method used to generate the random allocation sequence** Did the authors report the method of sequence generation (e.g., a random number table or computerized random number generator, or other)				
**Allocation concealment mechanism**	**Item 9. Mechanism used to implement the random allocation sequence** (e.g., sequentially numbered containers), describing any steps taken to conceal the sequence until interventions were assigned				
**Blinding**	**Item 11. Was the study blinded yes/no/not reported?** **-If yes go to 11a** **-If no or not reported, go to 13a** **Item 11a.** If done, who was blinded after assignment to interventions (e.g. participants, care providers, those assessing outcomes) and how blinding was performed (e.g. used of placebo) **Item 11b.** If relevant, description of the similarity of interventions (e.g., appearance, taste, smell, method of administration)				
**RESULTS**					
**Participant flow** *Comments:* *2: The flow-chart or text should clearly state how many participants got analyzed* *4: Only “no”, if there are clear signs that participants did not receive the allocated treatment* *5: flowchart/text should indicate that no participant stopped/discontinued the treatment* *6: if there is no indication for loss to follow-up, answer, “sufficient”, if clearly reported answer “yes”*	**Did the authors report a flow chart?** **Item 13a. For each group, were the following subitems reported** 1. Number of participants randomized in each group 2. Number of participants who received the intended treatment in each group 3. Number of participants analyzed for the primary outcome in each group **Item 13b. For each group, losses and exclusions after randomization, together with reasons** 1. Number of participants who did not receive the allocated treatment with reasons in each group 2. Number of participants who discontinued intervention with reasons in each group 3. Number of participants lost to follow-up with reasons in each group 4. Number excluded from analysis with reasons in each group				
**Outcomes and estimation**	**Item 17a. For each primary outcome, results for each group, and the estimated effect size and its precision** 1. Result in each group (mean (SD) or number of events/N) 2. Difference in estimated effect between groups (e.g., odds ratio (OR), risk ratio (RR), risk difference (RD), hazard ratio (HR), difference in median survival time, mean difference (MD)) 3. Precision for difference between groups (e.g., 95% CI)				
***Harms*** *Comments:* *If primary outcome(s) is the evaluation of harms, then here we will focus on the rest of harms or the globality of harms* *5. If there is no obvious dropout in the analysis, we expect no withdrawals due to harms*	**Item 19. All-important harms or unintended effects in each group and how they were reported** 1. List of adverse events with definition? (classification/grading, expected or not…) 2. Mode of data collection (Full description of methods used to collect the harm related information, who collected the information) 3. Timing (description of time frame of surveillance) 4. Attribution methods (i.e. “related” or not to treatment. Is the person responsible making attribution disclosed and whether blinding was used) 5. For each group, participant withdrawals due to harms 6. Results in each group for each type of harms with denominator (mean [SD] or number of events/N)				
**OTHER**					
Trial registration	**Item 23.** Registration number and name of registry				
**Consistency between data registered and reported in articles** Did authors report the same primary outcome in the registry and article (same variable, same metric, same time point) or was the primary outcome added, deleted, changed					
1. Was the primary outcome(s) reported in the registry or manuscript sufficiently described to identify a switch in outcome(s)? *	**Yes**		**No**		
2. **If yes, did you identify a switch in primary outcome(s)?** *Stop here if you have answered no to question 1*	**Yes**		**No**		
3. **Did you identify any outcome(s) reported by the authors as a primary outcome(s) but not registered as such?**	**Yes**		**No**		
4. **Did you identify any outcome(s) registered as a primary outcome(s) but not reported as such in the manuscript?**	**Yes**		**No**		
5. Did you identify any change in terms of time frame, metric or definition between the primary outcome(s) registered and reported in the article? *	**Yes**		**No**		
**6. If yes, please list the discrepancies:**					
-					
7. **Did the authors justify the switched outcome(s) in the manuscript?**		**Yes**		**No**	**NA**

^a^NA: non-assessable

^*^If the primary outcome is not clearly stated in the published article as such put “No”

In order to evaluate subitem 6a, our strategy for the choice of reported primary outcome was as follows:

-Look at the primary objective

-Look at the sample size calculation

-Look at the primary outcome stated in the registry

-If none of the above, chose the first one listed in the paper

#### Selective reporting

##### Retrospective registration on ClinicalTrials.gov

We assessed the percentage of trials with retrospective registration (trials that were registered after the trial start date).

##### Primary outcome(s) switching

*Identification of primary outcome(s) switching*

We searched for primary outcome(s) switching between the published full-text article and the ClinicalTrials.gov registry (Table 1). Primary outcome(s) switching was defined as adding or removing a primary outcome, or changing its definition (including changing, adding or removing the time frame or metric). Combination of more than one discrepancy were also considered (i.e. change in definition resulting in adding/removing a primary outcome). If the two primary outcomes differed because the registered primary outcome was more imprecise, we classified the trials as having “imprecise outcome registration” and not primary outcome(s) switching. For comparison of primary outcomes, we used the outcome(s) in the ClinicalTrials.gov registry labelled as “original primary outcome(s)” and not “current primary outcome(s)”, unless only a “current outcome(s)” was available. If the article did not mention a clear primary outcome, primary outcome switching could not be assessed.

*Evaluation of the effect of primary outcome(s) switching*

We also determined whether primary outcome(s) switching favored significant primary outcomes by applying the following strategy. From the full-text article, we extracted *p*-values for all outcomes reported in the article. We quoted results according to statistical significance: results significantly supporting or refuting the study intervention (or one of the groups in multi-arm trials) (i.e., *p* < 0.05), results that did not reach statistical significance (i.e., *p* ≥ 0.05), or unclear results. The same quoting was applied for equivalence or non-inferiority trials, according to the margin of equivalence set. A discrepancy was considered to favor significant results when a new statistically significant efficacy primary outcome was introduced or when a non-significant one was omitted or defined as secondary in the published article. We also considered a discrepancy as positive when a new, statistically non-significant safety primary outcome was introduced in the published article (e.g. if the experimental arm had no more adverse effects than the comparator even though the trial was not powered to show a difference). All the other cases were considered as negative discrepancies. The influence of some discrepancies could not be assessed because the article contained no results for the registered primary outcome or for the new primary outcome in the article (e.g. no summary measure for primary outcome described in the article). Similarly, the influence of discrepancies for “imprecise outcome registration” could not be assessed. For these cases, the influence was considered “Non-assessable”. Discrepancies were identified by one of us (A.P.) and confirmed with another member (I.B.).

### Statistical analysis

We used the R software (R studio Version 1.2.5033) for all analysis. Binary results were given in percentages.

## Results

### Sample identification

A total of 101 RCTs fulfilled our eligibility criteria and were evaluated for transparency and selective reporting.

Our dataset with results extracted for each included trial has been uploaded on Zenodo and is accessible with the following link https://doi.org/10.5281/zenodo.5841651.

### Transparency indicators

#### Access to trial documentation

About a third of trials in our sample (34, 34%) gave open access to the protocol (Table 2). It could be accessed through the published article (supplementary document or reference for a separate article), and/or the registry. In all cases the protocol was in English, and for 29 (29/34 = 85%) trials it was complete (i.e. not abbreviated or redacted). Finally, the statistical analysis plan was available for 32 (32%) trials.

**Table 2:** Mains results for transparency indicators for the 101 trials in our study

	**Number (N)**	**N/101 (%)**
**Access to trial documentation**		
Available protocol	34	34
*Access in publication*	20	20
*Access in registry*	8	8
*Both access*	6	6
Available statistical analysis plan	32	32
**Data sharing**		
Willing to share	25	25
*Statement in publication*	19	19
*Statement in the registry*	5	5
*Statement in both publication and registry*	1	1
Not willing to share	25	25
No statement on data sharing	51	50
**Completeness of reporting**		
Complete reporting of all items	5	5
Partial reporting of all items	96	95
Reporting of primary outcome(s) measure(s)		
*Complete*	74	73
*Partial*	27	27
Reporting of randomization and allocation concealment		
*Generation of the allocation sequence*	73	72
*Allocation concealment*	53	52
Reporting of blinding		
*Status unclear*	7	6
*Unblinded trials*	47	47
*Blinded trials*	47	47
Reporting of participant flow		
*Available flow chart*	94	93
*Complete*	81	80
*Partial*	19	19
*Not reported*	1	1
Reporting of results for the primary outcome(s)		
*Complete*	55	53
*Partial*	42	42
*Not reported*	4	5
Reporting of harms		
*Complete*	13	13
*Partial*	49	48
*Not reported*	39	39
Reporting of registration number	90	89

#### Data sharing statement

In our sample, 25 (25%) trials were willing to share data (Table 2). Of these, 22 (22/25 = 88%) gave information on where data could be accessed, including two with free access on the National Center for Biotechnology Information (NCBI). Information on the time frame of availability was specified by six trials (6/25 = 24%). Finally, 8 (8/25 = 32%) trials only agreed to share data with researchers and 14 (14/25 = 56%) had additional limitations (e.g. subject to approval). For the one trial where the data sharing statement was available in both the registry and publication, information was consistent. Finally, two trials (2%) did not agree to share their datasets while agreeing to share the protocol and/or statistical analysis plan and/or informed consent form.

#### Completeness of reporting (Tables 1 and 2)

Overall, only five trials (5%) reported all selected CONSORT items completely. Main results are summed up in Table 2.

##### Reporting of primary outcome(s) measure(s) (item 6a)

The primary outcome was not clearly identified in 12 (12%) trials, so a primary outcome was chosen for reporting (see Methodology section). For the 27 (27%) trials with partial reporting, the most frequently missing subitems were: description of the outcome assessor (20/27, 74%), and timing of outcome assessment (6/27, 22%).

##### Reporting of randomization and allocation concealment (items 8a and 9)

Most trials (73, 72%) specified the methods for the generation of the allocation sequence. The process of allocation concealment was reported by 53 (52%) trials.

##### Reporting of blinding (item 11)

Forty-seven (47%) trials were blinded. Among those, 32 (32/47 = 68%) described the blinding procedure completely. The most frequently missing subitems for completeness of reporting were description of how blinding was done in seven (7/47 = 15%) trials and who was blinded in four (4/47 = 9%).

##### Reporting of participant flow (items 13a, 13b)

Eighty-one (80%) trials provided a complete description of the participant flow, either as a diagram and/or in the main text. Trials with partial reporting commonly missed to report the number of patients who discontinued the trial intervention (12/19 = 63%).

##### Reporting of trial results for the primary outcome(s) (item 17a)

Among the 42 (42%) trials with partial reporting of primary outcome results, the effect size (e.g. odds ratio, hazard ratio, mean difference) and its precision were not reported in 34 (34/42 = 81%) and 38 (38/42 = 90%) trials respectively. Summary outcome result for each arm was missing in four (4/42 = 10%) trials.

##### Reporting of harms (item 19)

The majority of trials reported incomplete (49, 48%) or no information on harms (39, 39%). Regarding partial reports, the most frequently missing subitems were: the method for data collection (33/49 = 67%), the method of harms attribution (39/49 = 80%) and the timing of harms surveillance (24/49 = 49%). Among the 13 (13%) trials with complete reporting on harms, information was extracted from the protocol in 11 cases.

##### Reporting of registration related information (item 23)

Most trials reported the trial registration number in the published paper (90, 89%).

### Selective reporting

#### Retrospective registration

Among the 101 trials, 49 (49%) were retrospectively registered.

#### Primary outcome(s) switching

In our sample, 12 (12/101 = 12%) trials could not be assessed for primary outcome(s) switching in the absence of a clear primary outcome(s) in the article. Three (3/101 = 3%) trials had “imprecise outcome registration” and were not assessed for primary outcome(s) switching. Sixty-six trials (66/101 = 65%) trials reported primary outcome(s) as pre-defined in the trial registry and 23 (23/101 = 23%) trials had primary outcome(s) switching. Ten (10/23 = 43%) trials had an added or removed primary outcome between registration and publication, five (5/23 = 22%) trials had only a change of definition (including time frame or metric), and eight (8/23 = 35%) trials had a combination of change of definition and added or removed primary outcome. For the 23 trials with primary outcome(s) switching, the influence of the discrepancy could be evaluated in 16 (16/23 = 70%) trials. Among them, 6 (6/16 = 38%) trials has a discrepancy that favored statistically significant results. Finally, no trials with primary outcome(s) switching mentioned the change in the article.

## Discussion

Our work is the first to study transparency and selective reporting in a large sample of completed and terminated RCTs studying CRC management.

Our results showed little availability of trial protocols (34%), few positive sharing statements (25%) and rare completeness of reporting (5%). Important items or subitems often partially or not reported were harms, description of allocation concealment, information on the individual assessing the outcome and the number of patients who discontinued treatment in each trial arm. On top of that, almost half of trials were retrospectively registered and 23% had primary outcome(s) switching.

It has been shown in previous works that research transparency is crucial to allow for reproducibility of results and for the constitution of a strong body of evidence [7, 8]. Data sharing is important to evaluate the quality of the primary research which can impact future conclusions from reanalyzes of trials or from evidence synthesis research such as meta-analyses [8, 11, 12]. It can also help detect selective reporting used to make a trial “more publishable” (e.g. selective reporting of positive results). It was also shown that access to trial protocols helped detect selective outcome reporting [5, 13]. With the same goal of transparency, the 2010 CONSORT statement provides a minimum set of recommendations for reporting of RCTs. Previous works have shown that adherence of authors to these guidelines is low, which is in line with our findings [9–12]. Regarding selective reporting, about half of trials in our work were retrospectively registered. This could either mean that data is not always of high quality in registries [2] or that trials were voluntarily registered after the trials start date (all evaluated trials in our sample had a study start date after 2004 when registration became mandatory). For the identification of primary outcome(s) switching, we used the “original registered primary outcome” and not “current registered primary outcome”, considering that an update of the primary outcome on the registry after the study start date should have been mentioned in the article. Twenty-three % of trials in our work showed primary outcome(s) switching. Previous studies have also shown that outcome(s) switching between registry and publication is a frequent issue and that discrepancies sometimes favor statistically significant outcomes in the article [14, 15]. This was the case for 38% (6/16 = 38%) of concerned trials for which the influence of discrepancy was assessable. To our knowledge, similar work on assessment of transparency and selective reporting has not been performed for interventional research on other types of cancer. Therefore, it would also be interesting to perform this work in other frequent types of cancer, such as breast and prostate cancer, as well as in less frequent types of cancer, to see if prevalence impacts the overall reporting results.

Various tools have been developed to help for the reporting of clinical trials. For authors, an online writing aid tool for randomized trial reports, the CONSORT-based WEB tool (COBWEB tool), has helped improve completeness of reporting for RCTs [16]. Similarly, the COBPeer tool has been developed to help with the peer-review process of RCTs [9]. It was shown that trained early career researchers using the COBPeer tool were more likely to detect inadequate reporting (incomplete reporting or switch in primary outcome(s)) than the usual peer-review process used by journals [9]. Finally, reporting of RCTs can also be assessed through registries and not only the publication. For instance, one study found that trial results for RCTs studying drugs were more completely reported on the ClinicalTrials.gov registry than in the published articles [17]. Efforts need to be focused on reducing discrepancies between reports in registries and publications, but also improving the overall quality of reporting.

Our work has some limitations. First, by searching for RCTs through one registry we did not consider unregistered trials or trials registered on another registry. Also, the information on registries is not always of high quality. As an example, we found four trials with imprecise registration outcome. Then, we only evaluated 101 full-text published articles published over the past three years. We chose this time range for an optimal evaluation of recent articles since quality of reporting has improved over time following guidelines’ publication [18, 19]. Data sharing can lead to several challenges such as choosing the optimal sharing format, as well as the cleaning and interpretation of the original data for reanalyzes [8]. Furthermore, access to individual patient data is mainly useful for systematic reviews, and in particular, individual patient data meta analyses [20]. Finally, evaluation of reporting was mainly done by one reviewer and checked with a senior reviewer.

## Conclusion

In conclusion, there is a lack of transparency for evaluated RCTs with only few trials showing complete reporting of the selected CONSORT-items and/or willing to share data or study documentations. Also, selective reporting is frequently encountered. There is room for improvement.

## Supplementary Information

**Additional file 1.** Search strategy and eligibility criteria.

**Additional file 2.** Data extraction sheet.

**Additional file 3.** Data sharing explanation sheet.

## Data Availability

The datasets used and analyzed during the current study are available from the corresponding author on reasonable request.

## Abbreviations

CRC: Colorectal cancer
COBPeer tool: CONSORT based peer-review tool
COBWEB tool: CONSORT-based WEB tool
CONSORT: Consolidated Standards of Reporting Trials
GCO: World Health Organization Global Cancer Observatory
NA: Non-assessable
RCTs: Randomized controlled trials
US: United States
