# Supplementary material for: Assessment of transparency and selective reporting of interventional trials studying colorectal cancer
Source: BMC Cancer. 2022 Mar 15;22:278. doi: 10.1186/s12885-022-09334-5 (PMC8925077; doi:10.1186/s12885-022-09334-5)
Supplement: Supplementary file 1 — Additional file 1. Search strategy and eligibility criteria. [file 12885_2022_9334_MOESM1_ESM.docx]

**Additional File 1.** Search strategy and eligibility criteria

## Search strategy

We searched the United States (US) National Library of Medicine database of clinical trials, ClinicalTrials.gov, on the 11^th^ of March 2021 for all colorectal cancer (CRC) clinical trials performed in adults and completed or terminated over the past 7 years. We downloaded the list of encountered trials which were all referenced on the registry with a unique identification code or NCT number.

To identify full-text online publication of results for each trial, we used the publication link in ClinicalTrials.gov when available and posted. If a publication of results was found, the search was stopped. If no link was posted on the registry, we also systematically searched MEDLINE via PubMed and Google Scholar using keywords for treatment and/or drug names, the principal investigator’s last name and the condition studied. For industry-sponsored trials, we also searched the sponsor’s website via Google to look for the final results of industry-funded trials. We also used keywords for treatment and/or drug names and the condition studied on the sponsor’s website. If more than one publication of results was identified, we kept the publication of primary outcome(s) results as stated in the article. All trials without available published results were censored on April 15^th^, 2021.

## Eligibility criteria

Inclusion criteria for our sample were: all completed or terminated interventional trials, performed in adults (16 years old and over), randomized, focusing on CRC management, with a primary completion date between 01/01/2013 and 01/01/2020, and with results published online in a full-text article in English between 2021-03-22 and 2018-03-22.

Primary completion date was defined as the date on which the last participant in the trial was examined or received an intervention to collect final data for the primary outcome measure. Whether the clinical trial ended according to the protocol or was terminated did not affect this date. For clinical trials with more than one primary outcome measure with different completion dates, this term referred to the date on which data collection is completed for all the primary outcome measures.

We then excluded trials focusing on the wrong conditions (e.g. mixed malignancies, surgery of benign colorectal lesions, colonoscopy preparation or adenoma detection outside of CRC screening or cancer predisposition syndromes), feasibility or non-comparative trials and diagnostic test accuracy trials. We also excluded trials with results for which only an abstract, research letter or summary report was available, as well as nested trials and trials for which results were only published as a pooled analysis of multiple trials.

All identified publications were assessed by one reviewer who determined if 1) the corresponding trial matched in terms of the information registered (i.e., same NCT when mentioned, same studied condition, same interventions, same population, same trial location, same sponsor, same authors, and same time period) and 2) was a full-text article stating to report main trial results. The identification of eligible trials was done by one independent reviewer and checked with a second reviewer.
