# Supplementary material for: Assessment of transparency and selective reporting of interventional trials studying colorectal cancer
Source: BMC Cancer. 2022 Mar 15;22:278. doi: 10.1186/s12885-022-09334-5 (PMC8925077; doi:10.1186/s12885-022-09334-5)
Supplement: Supplementary file 2 — Additional file 2. Data extraction sheet. [file 12885_2022_9334_MOESM2_ESM.docx]

**Additional File 2**. Data extraction sheet

- 1. General Information

| **NCT Identification (ID)** |  |
| --- | --- |
| **Trial ID** *(surname of first author and year first full report of trial was published e.g. Smith 2001)* |  |
| **PMID** *(if pubmed search)* |  |
| **Published Title** |  |
| **Name of the journal** |  |
| **Date of online publication** *(YYYY-MM-DD)* |  |
| **Reference citation** *(doi)* |  |
| **Study author contact details** *(email)* |  |
| **Open access to article** | Yes  No |

- 1. Trial documentation

| **Data sharing statement: willing to share data?** | Yes  No  Not reported |
| --- | --- |
| **Where is the statement made?** | Registry  Publication  Both  NA |
| **If planned data sharing** *(yes to previous question)*  *Comment: see data sharing explanation sheet* | Content: “which data”, “other”  Process: “where”  Access point: give link or address  When  With whom  Requirements: “how”  Limitations |
| **Open available protocol** | Yes  No |
| **If available protocol** *(yes to previous question)* | Content: full, abbreviated or redacted  Language: English or other |
| **Open available statistical analysis plan(s)** | Yes  No |

- 1. Methods

|  | **Descriptions as stated in report/paper** | **Location in text or source** *(pg & ¶/fig/table/other)* |
| --- | --- | --- |
| **Primary outcome(s) used in publication** *(1 line per outcome)*  *See COBPeer tool* |  |  |
| **Identification of a switch of primary outcome possible?**  *See COBPeer tool* | Yes  No |  |
| **Did you identify primary outcome(s) registered but not published? or the other way around?**  *See COBPeer tool* | Yes  No  NA |  |
| **Did you identify any change in terms of time frame, metric or other information between the primary outcome(s) registered and reported in the manuscript?**  *See COBPeer tool* | Yes  No  NA |  |
| **If yes, specify which**  *See COBPeer tool* |  |  |
| **If yes, was the switch justified by authors?**  *See COBPeer tool* | Yes  No  NA |  |
| **Primary outcome(s) clearly identified?** | Yes  No |  |
| **Variable of interest clearly defined?** | Yes  No |  |
| **Clear definition of how the outcome was assessed? (measure)** | Yes  No |  |
| **Analysis metric?** | Yes  No |  |
| **Summary measure for each study group?**  *(depending on the type of outcome)* | Yes  No |  |
| **Time point for analysis** *(time of assessment; NA for survival analysis)* | Yes  No  NA |  |
| **Individual assessing the outcome clearly defined?** | Yes  No |  |
| **Randomization clearly described** | Yes  No |  |
| **Allocation concealment: method used to implement the random allocation sequence** *describing any steps taken to conceal until interventions were assigned* | Yes  No |  |
| **Baseline imbalance(s)** *(suggesting problem with randomization ?)* | Yes  No |  |
| **Blinding** | Yes  No  Not reported |  |
| **Blinding: who?** | Yes  No NA if no blinding |  |
| **Description of who was blinded** | Participant and/or investigator and/or outcome assessor and/or other…. |  |
| **Blinding: how?** *(e.g.: placebo)* | Yes  No NA if no blinding |  |
| **Blinding: If relevant, description of the similarities of interventions** | Yes  No  NA if no blinding |  |

- 1. Participant flow

|  | Description as stated in report/paper | Location in text or source *(pg & ¶/fig/table/other)* |
| --- | --- | --- |
| **Flow chart reported?** | Yes  No |  |
| **No. randomly assigned in each group?** | Yes  No |  |
| **No. who received the intended treatment?** | Yes  No |  |
| **No. who did not receive the intended treatment?** | Yes  No |  |
| **No. lost to follow up?** *(with reasons)* | Yes  No  Sufficient |  |
| **No. who discontinued the intervention?** *(& whether the reasons were specified). NA for screening trials* | Yes  No  NA |  |
| **No. of patients analysed for the primary outcome?** *(& whether the reasons were specified)* | Yes  No |  |
| **No. of patients excluded from analysis?** *(& with reasons)* | Yes  No |  |
| **ITT analysis done?** | Yes  No |  |

- 1. Outcomes and estimations

For each primary outcome, results for each group, estimated effect size and its precision

| **Results in each group?** *(mean or number of events)* | Yes  No |  |
| --- | --- | --- |
| **Difference in estimated effect between groups?** | Yes  No |  |
| **Precision for difference between groups?** | Yes  No |  |

- 1. Harms (adverse events and serious adverse events)

|  | Description as stated in report/paper | Location in text or source *(pg & ¶/fig/table/other)* |
| --- | --- | --- |
| **List and clear definition** **of harms?** *(adverse events and serious adverse events)* | Yes  No |  |
| **Description of mode of data collection?** | Yes  No |  |
| **Timing?** *(description of timeframe and surveillance)* | Yes  No |  |
| **Attribution methods?** | Yes  No |  |
| **No. of withdrawals due to harms?** | Yes  No |  |
| **Results in each group for each harm with denominator?** | Yes  No |  |

- 1. Other information

|  | **Description as stated in report/paper** | **Location in text or source** *(pg & ¶/fig/table/other)* |
| --- | --- | --- |
| **Registration number for the trial in publication?** | Yes  No |  |
| **Conflicts of interest (COI) statement?** | Yes  No |  |
| **Funding statement?** | Yes  No |  |
| **Type of funding** | Industry-related [12] Non-industry related  Mix |  |
