# Supplementary material for: Assessment of transparency and selective reporting of interventional trials studying colorectal cancer
Source: BMC Cancer. 2022 Mar 15;22:278. doi: 10.1186/s12885-022-09334-5 (PMC8925077; doi:10.1186/s12885-022-09334-5)
Supplement: Supplementary file 3 — Additional file 3. Data sharing explanation sheet. [file 12885_2022_9334_MOESM3_ESM.docx]

**Additional file 3**. Data sharing explanation sheet

**Data sharing explanation sheet for extraction:**

**data_sharing_willing:** Are authors willing to share data (*look at both registry and publication*)?

Answers should be “Yes” or “No”. If no information on data sharing in the paper or in the registry, we go for “not reported”

**data_statement_where:** Where can you find the data sharing statement?

- Registry
- Publication
- Both

**If the answer is “Not reported” or “No” for the first item, the rest of the columns should be filled with “Non-assessable (NA)”. Otherwise, please answer the following questions:**

**data_sharing_which:** Specify which kind of data is shared.

- Complete data
- Data underlying article (*comment: Only the data that was presented in the article*)
- Data without specification
- Other
- Not reported

**data_sharing_other:** Will other documents be available, if not in open access (trial protocol, statistical analysis plan, informed consent form)

- Yes
- No
- Not reported

**data_sharing_where:** Specify how data can be accessed.

- Author/Other contact
- Available online
- Sponsor
- Not reported

**data_sharing_access_point:** Add reported link/email address or any other kind of information regarding this point

**data_sharing_when**: When does the trial plan to share data? Compare with publication date to answer, if only date is given.

- After trial completion
- No delay after publication
- Delay after publication
- Not reported

**datasharing_with_whom (restrictions):** Can anyone get access to data or only certain persons?

- Researcher
- Anyone
- Other *(e.g. journal)*
- Not reported

**data_sharing_requirements:** What process needs to be done to get access to the data?

- Access enquiry form (*comment: meaning that a formular is needed*)
- Author/Other contact (*comment: In this case if not specified, this point is a repetition of data sharing_where*)
- Statement of intention (*comment: Intention and inquiry without specific format*)
- No requirement
- Not reported

**data_sharing_limitations:** Can access be refused?

- No
- Subject to approval
- Other (*ex: under reasonable request, through discussion….*)
- Not reported
